# The assessment of inflammatory activity and toxicity of treated sewage using RAW264.7 cells

**DOI:** 10.1111/wej.12127

**Published:** 2015-06-09

**Authors:** Vedastus W. Makene, Edmund J. Pool

**Affiliations:** ^1^ Department of Medical BioscienceUniversity of the Western CapeBellville7535South Africa

**Keywords:** effluent, environmental assessment, pollution, sewage, wastewater

## Abstract

Toxicity and inflammatory activity of wastewater samples were evaluated using RAW264.7 cells as a bioassay model. The RAW264.7 cell cultures were exposed to sterile filtered wastewater samples collected from a sewage treatment plant. Cell viability was evaluated using WST‐1 and XTT assays. Inflammatory effects of samples were assessed by determination of nitric oxide (NO) and interleukin 6 (IL‐6). The NO was estimated using the Griess reaction and IL‐6 was measured by enzyme‐linked immunoassay. All samples had no toxicity effects to RAW264.7 cells, however they significantly (*P* < 0.001) induced NO and IL‐6 production. The highest NO (12.5 ± 0.38 μM) and IL‐6 (25383.84 ± 2327 pg/mL) production was induced by postbiofiltration sample. Final effluent induced the lowest inflammatory response, which indicates effective sewage treatment. In conclusion, wastewater samples can induce inflammatory activities in RAW264.7 cells. The RAW264.7 cells, therefore, can be used as a model for monitoring the quality of treated sewage.

## Introduction

Sewage is normally composed of many types of pollutants. Raw sewage may contain high levels of pollutants like nutrients, inorganic chemicals, organic micropollutants, microorganisms and microbial products like endotoxins (Carlson *et al*. 2013; da Silva *et al*. 2013). When inadequately treated, it can pollute water bodies. In municipal effluents, common pollutants are microbes, heavy metals, steroids, pharmaceuticals, personal care products, industrial and domestic chemicals (Naidoo and Olaniran 2013; Du *et al*. 2014; Faul *et al*. 2014). Discharged municipal effluents can, therefore, be a source of contamination to receiving surface water and drinking water (Stackelberg *et al*. 2004; Naidoo and Olaniran 2013). The contaminated water has been associated with many health problems including immunotoxicity (Gust *et al*. 2013). Many of the pollutants in wastewater can be immunotoxic because of the induction of inflammatory reactions (Wichmann *et al*. 2004; Xu *et al*. 2013; Kim *et al*. 2014).

The inflammatory reaction is one of the defence mechanisms of the immune system, which is induced by the presence of pathogens. The first line of defence system is innate immune system, which is mediated by phagocytes such as neutrophils and macrophages (Newton and Dixit 2012). When macrophages are activated, they initiate an inflammatory response by producing a wide range of inflammatory mediators and proinflammatory cytokines (Newton and Dixit 2012). Common inflammatory mediators and proinflammatory cytokines secreted by macrophages includes nitric oxide (NO), interleukin 1 (IL‐1), interleukin 6 (IL‐6) and tumour necrosis factor alpha (TNFα) (Barnes *et al*. 2011). IL‐6 is a proinflammatory cytokine with many functions (Mihara *et al*. 2012). In inflammatory reactions, IL‐6 induces acute phase responses and also supports chronic inflammation processes (Barnes *et al*. 2011; Mihara *et al*. 2012).

The proinflammatory cytokines are also known for induction of inducible nitric oxide synthase (iNOS), a key enzyme in NO production. The iNOS enzyme catalyzes production of NO from l‐arginine. The NO produced is a highly reactive molecule important for cell signalling and killing of pathogens (Omer *et al*. 2012). Therefore, NO is normally used as a biomarker of inflammation. Unfortunately, NO has a very short half‐life, hence excess is instantly converted and stored in the form of nitrite (Shiva 2013). The level of nitrite produced is, therefore, used in many studies to estimate NO production (Liu *et al*. 2012; Kim *et al*. 2014).

The production of NO and IL‐6 has been used as biomarkers of inflammatory responses in many studies. There are *in vivo* studies which used NO and IL‐6 as biomarkers of inflammatory reaction (Avdagić *et al*. 2013; Piva *et al*. 2013). Increased production of NO is a known biomarker of inflammatory response in the respiratory system (McCluskie *et al*. 2004). Therefore, exhaled NO has been used to monitor respiratory inflammation associated with air pollutants (Berhane *et al*. 2014). Similarly, increased secretion of IL‐6 has been also reported to be a sensitive biomarker of inflammatory activity (Pool *et al*. 2000). In fact, there is an increase of studies using *in vitro* assays for IL‐6 as a biomarker of inflammatory. For instance, there has been extensive use of whole human blood culture to detect IL‐6 as a biomarker for inflammatory responses (Pool *et al*. 2003; Pool and Magcwebeba 2009; Faul *et al*. 2014). Others are using isolated peripheral human mononuclear cell culture (Wichmann *et al*., 2004; Adebayo *et al*., 2014).

The use human blood culture for routine monitoring of inflammatory activities in water may be hindered by ethical issues. Alternative to using human blood culture, the inflammatory response can be evaluated using established cell lines. One of the most widely used cell lines in inflammatory studies is mouse macrophages like RAW264.7 cells. The RAW264.7 cells are from mouse ascites leukaemia‐induced cells. Once activated, RAW264.7 cells express inflammatory mediators and proinflammatory cytokines such as NO and IL‐6, respectively (Xu *et al*. 2014; Kim *et al*. 2014). As a result the cells have been used widely in many inflammatory response studies. For example, macrophage RAW264.7 cells have been used in studying the inflammatory effects of environmental *Mycobacterium* spp. (Huttunen *et al*. 2000). The RAW264.7 cells have been also used in inflammatory effects of air pollutants (Chauhan *et al*. 2004). Furthermore, the cell line has been used extensively for testing of inflammatory and anti‐inflammatory effects of natural products (Szliszka *et al*. 2013; Xu *et al*. 2014; Yang *et al*. 2014).

Despite extensive literature searches, no research report on the use of RAW264.7 cells for testing inflammatory activities of water or wastewater was found. In this study, we have evaluated the effects of wastewater samples on RAW264.7 cells. This study focused on the cytotoxicity and inflammatory activity of these samples to determine if the RAW264.7 cell line is a sensitive model for sewage quality monitoring.

## Material and methods

### Sewage water samples

**Figure 1:**
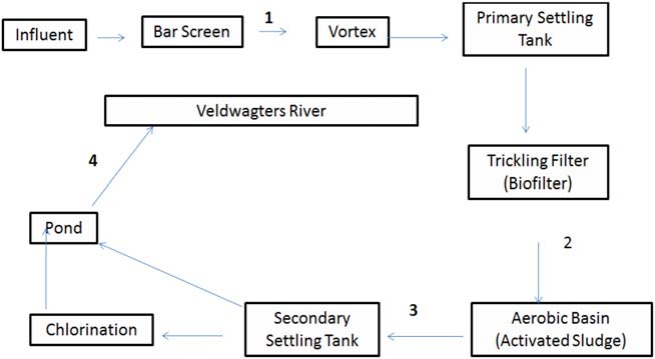
Stellenbosch wastewater treatment works (SWTW) layout: showing wastewater treatment stages and water sample collection points. 1‐Influent sample, 2‐Postbiofilter sample, 3‐Postactivated sludge sample and 4‐Final effluent sample.

Water samples were collected from Stellenbosch wastewater treatment works (SWTW) in Cape Town, South Africa. The processes involved at the plant are shown in Fig. 1. Water samples were collected at four different points, namely influent (1), postbiofilter (2), postsludge (3) and final effluent (4). Water samples were collected in clean glass bottles. An aliquot of each sample was immediately checked for total coliforms and *Escherichia coli*. Another aliquot was sterile filtered using 0.45‐μM filters and stored in sterile tubes at −20°C till used in cell culture. The rest of the water sample was used immediately for C‐18 extraction using DS‐18 solid phase extraction column (Supelco, Sigma, Germany). The extract was analysed for steroid concentrations. The steroid concentration and bacteriology measurement were carried out as a routine checks for plant efficiency. Total coliforms and *E. coli* were determined using premade ReadyCult coliforms medium (Merck, Germany). Steroids concentrations were determined using commercial available enzyme‐linked immunoassay (ELISA) kits (DRG, Germany). The uses of ELISA kits for determination of levels of steroids in water have been previously validated (Swart and Pool, 2007). The minimum detection limits of the assay are: estrone 2.21 pg/mL, estradiol 9.7 pg/mL, ethinyloestradiol 0.1 ng/L, progesterone 0.45 ng/mL, and testosterone 83 pg/mL. Samples were diluted accordingly with diluted wash buffer; and assay procedures provided in the kits were followed. The performance efficiency of the plant based on bacteriological and steroids concentrations are summarized in Tables 1 and 2, respectively.

**Table 1:** Bacteriological quality of water samples as determined by concentration of total coliforms and *E. coli* at different stages treatment in Stellenbosch STW

Sample	Total coliforms (cfu/mL)	*E. coli* (cfu/mL)
Influent	>1000	>1000
Postbiofilter	>1000	>1000
Postsludge	>1000	>1000
Effluent	<1	<1

cfu, colony forming units; mL, millilitre

**Table 2:** Concentration of steroid hormones in water samples (*N* = 4) from SWTW (Booysen, 2014)

	Estrone		Estradiol		Ethinylestradiol		Progesterone		Testosterone	
Sample ID	Conc (pg/mL)	SD	Conc (pg/mL)	SD	Conc (pg/mL)	SD	Conc (pg/mL)	SD	Conc (pg/mL)	SD
Influent	371.26	4.85	192.08	13.69	49.65	15.66	209.82	13.0	181.19	2.57
Postbiofilter	369.54	0.80	145.55	14.04	55.61	12.12	779.64	9.33	153.05	3.90
Postsludge	117.54	4.25	12.23	1.28	5.63	1.17	10.25	1.41	7.23	0.09
Final Effluent	203.80	18.81	29.62	1.55	10.78	1.11	12.58	0.00	6.87	1.19

### Cell culture

Mouse macrophage RAW264.7 cell line ATCC‐TB‐71 was cultured in Dulbecco's Modified Eagle's medium supplemented with 10% heat inactivated foetal bovine serum, 1% antibiotic/antimycotic (Sigma), 0.05% gentamycin (Sigma), and 1% glutamax, at 37°C and 5% CO_2_. The cells were cultured in 96 well plates at density of 5 × 10^5^ cells/mL till were almost confluent. At confluent cell culture were treated as follows: normal medium for negative control, medium supplemented with 1 μg/mL lipopolysaccharides (LPS) from *E. coli* 0111:B4 (Sigma) as positive control, medium containing sewage samples at 1 in 10 and 1 in 100 dilutions in respective wells. After overnight incubation at 37°C and 5% CO_2_, culture supernatants were collected for NO and IL‐6 assays. The cells on a plate were used for cell viability assays.

### Cell viability

Cell viability assays were determined using chromogenic‐based water‐soluble tetrazolium salts WST‐1 (2‐(4‐Iodophenyl)‐3‐(4‐nitrophenyl)‐5‐(2,4‐disulfophenyl)‐2H‐tetrazolium) and XTT (2,3‐bis‐(2‐methoxy‐4‐nitro‐5‐sulfophenyl)‐2H‐tetrazolium‐5‐carboxanilide) assays. The assays were performed using XTT reagent mixture (Roche, Germany) and WST‐1 reagent (Roche). The assays procedures were followed according to manufacturer's protocol provided.

### Nitric oxide

Secretion of NO was determined in culture supernatants using the Griess reaction in 96‐well plates (Nunc, Denmark). The supernatant was mixed with equal volume of Griess reagent made up of 1% sulphanilamide (Sigma), 0.01% naphthy ethylenediamine dihydrochloride (Sigma), and 2.5% phosphoric acid. The colour developed after 15 min incubation was measured at 540 nm using a microplate reader (Thermo electron). The concentration of NO was determined from a standard curve generated using 100 μM–1.56 μM sodium nitrite (Sigma).

### Interleukin 6

Interleukin 6 was determined in cell culture supernatant using a commercially available mouse IL‐6 ELISA kit (e‐Bioscience, Germany). The assay is a double antibody sandwich enzyme‐linked immunosorbent assay (DAS ELISA). The assays were performed on Nunc Maxisorp 96‐well plates (Nunc). All reagents and assay diluent were provided in the kit; and the assay kit's assay procedure was followed accordingly.

### Statistical analysis

The data are presented as mean ± standard deviation (SD), which were statistically analysed with one way variance analysis (ANOVA) using sigmastat (SigmaStat software, Inc., CA). The mean values of each treatment were compared with control. Sample size for all assays was four. The *P*‐value <0.001 was considered as statistically significant.

## Results

### Cell viability

**Figure 2:**
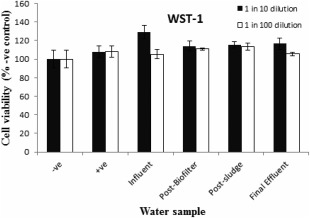
Effects of water samples on RAW264.7 cells viability as determined by WST‐1 assay. Negative control was treated with normal medium, positive control treated with LPS (1 μg/mL) and sample treatment at 1 in 10 and 1 in 100 dilutions.

**Figure 3:**
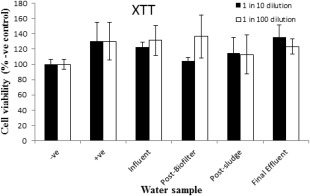
Effects of water samples on RAW264.7 cells viability as determined by XTT assay. Negative control was treated with normal medium, positive control treated with LPS (1 μg/mL) and water samples treatment at 1 in 10 and 1 in 100 dilutions.

Water samples at 1/10 and 1/100 dilutions in the culture medium were tested for effects on cell viability. All data were compared to the negative and LPS stimulated culture controls. The XTT and WST‐1 results for all the samples were similar indicating that the samples had no toxic effects to RAW264.7 cell viability (Figs. 2 and 3).

### Effects of water samples on NO production

**Figure 4:**
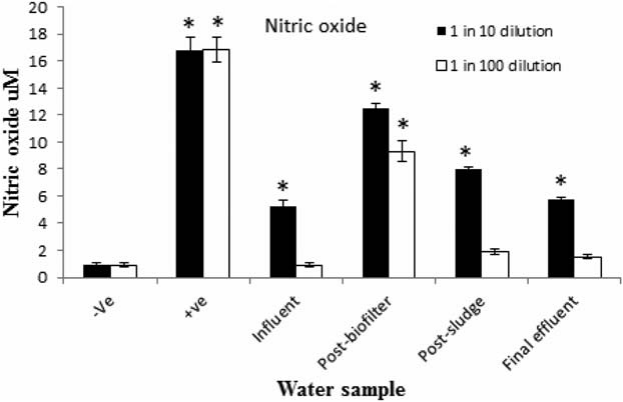
Effects of water samples on NO production in RAW264.7 cells. Negative control received normal medium alone, positive control was treated with LPS (1 μg/mL), water samples tested at 1 in 10 and 1 in 100 dilutions. * indicates that NO level is significantly (*P* < 0.001) higher than the negative control.

All water samples at 1 in 10 dilution increased production of NO significantly (*P* < 0.001) higher than the negative control (Fig. 4). The highest effect on NO production was induced by postbiofilter sample (12.5 ± 0.38 μM). The postsludge sample induced relatively decreased effect, while the lowest induction was evident with influent (5.2 ± 0.47 μM) and final effluent samples (5.7 ± 0.21 μM). Among the water samples at 1 in 100 dilutions, postbiofilter sample induced significantly (*P* < 0.001) higher NO production compared to the negative control.

### Effects of water samples on IL‐6 secretion

**Figure 5:**
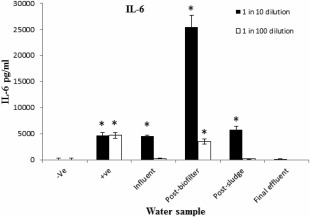
Effects of water samples on IL‐6 secretion in RAW264.7 cells. Negative control received normal medium alone, positive control was treated with LPS (1 μg/mL), water samples treatment at 1 in 10 and 1 in 100 dilutions. * indicates that the IL‐6 induced by the treatments is significantly (*P* < 0.001) higher than the negative control.

Water samples from influent, postbiofilter, and postsludge at 1 in 10 dilution significantly (*P* < 0.001) induced production of IL‐6 in RAW264.7 cells compared to the negative control (Fig. 5). The postbiofilter water sample induced the highest secretion of IL‐6 (25 383.84 + 2327 pg/mL). Induction of IL‐6 by effluent sample was similar to the negative control in both dilutions. Among all water samples tested at 1 in 100 dilutions postbiofilter sample induced significantly (*P* < 0.001) higher production of IL‐6 than the negative control.

## Discussion

Detection of pollutants in wastewater using chemical methods is a common technique of assessment of pollutants. However, because of the complex mixture of pollutants in wastewater, analysis of each pollutant is not easy. Alternatively, biological assays are usually developed for assessment of biological effects of wastewater samples. In this study, wastewater samples from SWST were assessed for toxicity and inflammatory activities using RAW264.7 cells. None of the water samples tested resulted in significantly different WST‐1‐ or XTT‐based metabolic assay compared to controls, which implies that samples had no toxic effects to RAW264.7 cells. Lack of toxicity of wastewater or contaminated water has been reported in previous studies (Pool *et al*., 2000; Hendricks and *Pool* 2012). This observation may be attributed to lower concentration of pollutants required to induce immunotoxicity as compared to that required for induction of cell death (Hymery *et al*. 2014).

Stimulation of RAW264.7 cells induces production of NO and other proinflammatory cytokines including IL‐6 (Liu *et al*. 2012; Kim *et al*. 2014; Xu *et al*. 2014). In this study, all samples at concentrations of 1 in 10 dilution induced NO production in RAW264.7 cells significantly (*P* < 0.001) higher than negative control. Similar inflammatory response was observed in the induction of IL‐6 secretion because of influent, postbiofilter and postsludge samples. The increase of NO and IL‐6 production in this study show that the water samples induced inflammatory responses in RAW264.7 cells. The induction of IL‐6 secretion has been reported as a sensitive biomarker for monitoring inflammatory activities of water (Pool *et al*. 2000). As a result of this, it has been used as a biomarker assay for water quality. Indeed, IL‐6 has now been used in many water assessment studies using human blood culture (Pool *et al*. 2003; Pool and Magcwebeba, 2009; Adebayo *et al*. 2014; Faul *et al*. 2014).

The results also show that the highest inflammatory activity in RAW264.7 cells was induced by postbiofilter sample. The sample induced significantly (*P* < 0.001) higher production of both NO and IL‐6 than the rest of water samples. The high levels of NO and IL‐6 is an indication of increased inflammatory activities because of postbiofilter water sample. The inflammatory effects of this sample could be associated with the presence of high content of inflammatory pollutants. A number of organic pollutants common in wastewater can induce inflammatory activities (Xu *et al*. 2013; Kim *et al*. 2014). The increase in inflammatory responses in postbiofilter sample can also be associated with high levels of steroids in this sample (Table 2). Some studies showed that estradiol can induce proinflammatory cytokines IL‐6 and iNOS through oestrogen receptor (ER_α_) and ER_β_ in macrophages (Calippe *et al*. 2010). Another possible source of high inflammatory activities in the postbiofilter sample could be associated with presence of microbial products like endotoxins because of high microbial activities in the biofilter process. The process in trickling filters is dominated by high microbial activities.

The results further show that influent water sample induced relatively low inflammatory responses as determined by NO level. The influent sample is normally a rich mixture of pollutants. Some of the pollutants in influent may have anti‐inflammatory effects. This sample has a high concentration of steroid hormones. Some steroids present in the water sample like progesterone and testosterone can suppress inflammatory activities (Rettew *et al*. 2008; Corcoran *et al*. 2010). Progesterone can suppress production of IL‐6 and NO as well as expression of iNOS and NF‐kB in macrophages (Su *et al*. 2009). Therefore, the low inflammatory response induced by the influent might be because of contaminants with anti‐inflammatory activities in the sample.

The final effluent sample induced relatively low inflammatory response, characterized by low level of NO and undetectable IL‐6 secretion. This water sample was collected at the postchlorination point just before discharged into a receiving river. The low inflammatory activities of the sample from this point could be because of effects of disinfection performed by chlorination at the preceding process. The results show that the treatment processes at SWTW remove most of the inflammatory pollutants. The findings in this study correlate well with previous studies using whole human blood culture in the assessment of water quality (Pool *et al*. 2003; Pool and Magcwebeba 2009; Faul *et al*. 2013). Similar trends of results have also been reported in studies using isolated human peripheral mononuclear cell culture (Wichmann *et al*. 2004; Adebayo *et al*. 2014). In order to avoid ethical issues surrounding uses of human blood, the induction of inflammatory responses in RAW264.7 cells observed in this study, therefore, provides an alternative bioassay model to using of human blood culture.

In conclusion, water samples collected from SWTW did not induce cytotoxic effects to macrophage RAW264.7 cells. However, the same samples induced inflammatory responses by increasing NO production and IL‐6 secretion in RAW264.7 cells. The increase of NO production and IL‐6 secretion in RAW264.7 cells can be used as biomarkers for inflammatory activity and monitoring water quality. The results also give evidence that RAW264.7 cells can be used as a model for monitoring inflammatory pollutants in water and wastewater in particular.
